# Plaque scores after 1 or 2 minutes of toothbrushing A systematic review and meta‐analysis

**DOI:** 10.1111/idh.12840

**Published:** 2025-04-08

**Authors:** Marion T. Seuntjens, Tim M. J. A. Thomassen, Fridus (G. A.) Van der Weijden, Dagmar Else Slot

**Affiliations:** ^1^ Department of Periodontology, Academic Centre for Dentistry Amsterdam (ACTA) University of Amsterdam and Vrije Universiteit Amsterdam Amsterdam The Netherlands

**Keywords:** brushing exercise, duration, minutes, plaque, single‐use, toothbrush

## Abstract

**Aim:**

To evaluate the difference in plaque score reduction achieved by 1 and 2 min of toothbrushing, based on data from single brushing exercises.

**Methods:**

MEDLINE‐PubMed, Cochrane‐CENTRAL databases and the first five pages of Google Scholar were searched for randomised controlled trials. Extracted data were summarised in a descriptive and, if possible, a meta‐analysis using standardised mean difference (SMD). Separate analyses were performed for manual (MTB) and powered (PTB) toothbrushes.

**Results:**

Based on the selection criteria, the search identified five unique eligible publications providing 16 comparisons. For both toothbrush types, 2 min of brushing resulted in a significantly higher plaque score reduction. The SMD of 1 versus 2 min of brushing using MTB was 0.69 [95% CI: 0.06, 1.33] (*p* = 0.03) can be interpreted as a medium size effect and the SMD of using a PTB was 0.47 [95% CI: 0.28, 0.66], (*p* < 0.00001) interpreted as a small size effect.

**Conclusion:**

With respect to plaque scores, based on single brushing exercises, there is moderate certainty for the recommendation to brush for 2 min over 1 min.

## 1 INTRODUCTION

It is estimated that oral diseases, notably periodontal diseases and caries, affect nearly 3.5 billion people. Globally, untreated dental caries is the most prevalent oral condition, affecting 2.3 billion people, whereas severe periodontal disease is estimated to affect 267 million people (WHO).^1^ Dental plaque is a microbial biofilm associated with caries and periodontal diseases.^2^ It is naturally found on teeth and is considered the most important causal factor for both conditions. Caries and periodontal diseases are unlikely to occur without dental plaque; when biofilm is adequately removed, there is little risk of it becoming hazardous.^3^, ^4^, ^5^ Thus, daily home care to remove dental plaque is presumably sufficient to prevent both caries and periodontal diseases. Moreover, proper oral hygiene habits might resolve gingivitis and prevent the progression of periodontal diseases.^6^ Mechanical tooth cleaning by brushing remains the most effective method of removing bacterial plaque,^7^ irrespective of whether dentifrice is used.^8^

The average plaque score reduction following a single brushing exercise using manual toothbrushes is 42%^9^ and 46% using powered toothbrushes.^10^ Two minutes of twice daily brushing is the standard expert advice of dental care professionals, dental care organizations and the oral hygiene industry.^11^, ^12^ Even though all dental stakeholders have propagated this standard, people brush on average approximately half of the advised 2 min.^11^, ^13^, ^14^ Research has indicated a correlation between brushing duration and the amount of plaque removal. As the brushing duration increases, more plaque is removed.^11^, ^13^, ^15^ Based on single brushing exercises, this finding is supported by sub‐analyses in systematic reviews.^9^, ^10^ For manual toothbrushes (MTB), an average 27% plaque score reduction occurs after 1 min, and a 41% reduction occurs after 2 min.^9^ For powered toothbrushes (PTB), the plaque score reduction depends on the plaque index used, but on average, the reduction is either 32% after 1 min and 38% after 2 min or 61% after 1 min and 67% after 2 min dependent of the plaque index used.^10^ These results represent indirect outcomes based on single legs from clinical trials and may not be representative for a direct head‐to‐head comparison of 1 and 2 min of toothbrushing. Considering the indirect evidence, direct comparisons in a clinical study would provide more insight. The results can support the clinical recommendations dental care professionals make to their patients and can be useful to other dental stakeholders, professional dental associations and textbooks. This systematic review aimed to compare the effect of 1‐ and 2‐min brushing duration on plaque scores based on single brushing exercises with adult participants who brushed their teeth themselves.

## 2 MATERIALS AND METHODS

### 2.1 Protocol and guidelines

This systematic review and meta‐analysis were prepared and described in accordance with the Cochrane Handbook for Systematic Reviews of Intervention and the Transparent Reporting of Systematic Reviews and Meta‐Analyses (PRISMA statement 2020).^16^ The protocol detailing the review method was developed a priori following an initial discussion among the research team members and was registered with the International Prospective Register of Systematic Reviews (*PROSPERO*)^17^ under registration number CRD42022298269 and the institutional review board of the Academic Centre for Dentistry Amsterdam (ACTA) under registration number 2021–49626 (Appendix S1). The crucial steps in the review process—screening and selection for eligibility, quality assessment, estimation of risk of bias, data extraction and grading the body of evidence—were performed independently by two reviewers using predefined procedures and forms (MS&TT). Disagreements were resolved by consensus, and if a disagreement persisted, a third reviewer's judgement (DES) was considered decisive.

### 2.2 Focused question

Based on studies using single toothbrushing exercises, What is the effect on plaque scores between 1 and 2 min of brushing?

The focused question was derived from the Patient, Intervention, Comparison, Outcome and Study design (PICOS) format^18^:

**Table:** 

Patient:	Patient using a toothbrush
Intervention:	1 min
Comparison:	2 min
Outcome:	plaque scores using, for instance but not limited to, Quigley and Hein plaque index (Q&HPI),^19^ or Turesky,^20^ Navy plaque index,^21^ Rustogi‐modified Navy plaque index (RMNPI),^22^ and Silness & Loë,^23^ Addy et al. (1983)^24^ modification of Shaw & Murray (1977)^25^
Studies:	Single‐use brushing exercises

### 2.3 Search strategy

A structured search strategy was designed to retrieve all relevant published scientific studies from peer‐reviewed sources that evaluated single brushing exercises to compare the results of 1 and 2 min of brushing durations. The National Library of Medicine, Washington, D.C. (MEDLINE‐PubMed) and the Cochrane Central Register of Controlled Trials (CENTRAL) were searched from their inception to April 2023 and the first five pages of Google Scholar^26^ (Scholar. google) until April 2023 for appropriate papers that answered the focused question. The reference lists of the included studies were also hand‐searched to identify additional potentially relevant studies. For details regarding the search terms used, see Table 1.

**TABLE 1:** Terms used for the search strategy.

{[subject] AND [intervention] AND [time]} {[<subject **Toothbrush** > Textwords: toothbrush OR toothbrushing OR toothbrush* OR MesH: Toothbrushing] AND [<intervention **Plaque** > Textwords: (Plaque removal) OR (plaque index) OR (dental plaque) OR plaque OR (interdental Plaque) OR (interproximal plaque) OR MesH: dental plaque] AND [<**Time/minute**> Time OR Minute OR Minute* OR Second OR second*]}

The asterisk (*) was used as a truncation symbol.

### 2.4 Screening and selection

The titles and abstracts were screened to select studies that potentially met the inclusion criteria. No language restrictions were imposed. The full‐text versions of potentially relevant papers were obtained and were categorised as definitely eligible, definitely not eligible or questionable. The papers that fulfilled the inclusion criteria were processed for data extraction.

The inclusion criteria were as follows:

- Randomised controlled clinical trials (RCTs) or controlled clinical trials (CCTs)
- Studies using a single toothbrushing exercise with participants using manual (MTB) or powered (PTB) toothbrushes by themselves (i.e. no professional brushing)
- Comparison of the same brand and type of toothbrush
- Conducted on humans
  - ≥18 years of age
  - In good general health (without systemic disorders)
- Minimum of 20 evaluable teeth
- An evaluation of 1 and 2 min of brushing (irrespective of what was considered the intervention or the control regimen in the retrieved study)
- Plaque index score assessments according, but not limited to, one of the following commonly used plaque indices or modified versions of them:
  - The Quigley and Hein plaque index (Q&HPI)^19^ or the Turesky modification^20^ assessed at two sites per tooth (or according to the Lobene^27^ modification of up to six sites per tooth)
  - The Navy Plaque Index^21^ or the RMNPI^22^
  - The Silness & Löe plaque index (S&LPI)^23^

The exclusion criteria were as follows:

- Pregnancy
- Orthodontic fixed appliance
- Removable prosthesis
- Dental implants
- Hospitalised or institutionalised.

### 2.5 Risk of bias assessment

The methodological quality of the included studies was scored using the Cochrane risk of bias tool.^28^ The scoring was based on seven domains and could be scored as unclear, low risk of bias or high risk of bias. The evaluated items included random sequence generation, allocation concealment, the blinding of participants and personnel, the blinding of the outcome assessment, incomplete outcome data and selective reporting and ‘others’. It was decided a priori that the domain of performance bias was not included in the overall estimation of risk of bias. This decision was based on the fact that although the included studies evaluated different brushing durations (1 min and 2 min), the participants could easily discern the difference. Consequently, blinding the participants or personnel so that they were unaware of the duration of brushing was impossible. The consequence of removing performance bias from a risk‐of‐bias consideration, the criterion for the overall estimation of the potential risk of bias, was that a study was estimated to be at a high risk of bias if at least one domain had a high risk of bias, at unclear risk of bias if at least one domain was unclear and none were high and at a low risk of bias if all domains were at a low risk of bias.^29^

### 2.6 Assessment of clinical and methodological heterogeneity

The data from the publications that met the selection criteria were extracted and processed for further analysis. The factors used to evaluate the heterogeneity of the outcomes of the different studies were as follows:

- Study design details
- Subjects' characteristics
- Study regimen and device (MTB or PTB)
- Funding and conflict of interest

### 2.7 Data extraction

The characteristics from all the included studies, the population data, the intervention and the comparison and outcomes were extracted independently to predefined forms. Only the data of those legs representing the 1‐ and 2‐min brushing duration were considered. Data related to other brushing durations were not extracted. For studies with multiple outcomes, for instance, two different plaque scores, and for which data were available for both the intervention and control groups, the number of participants (*N*) in the groups was divided by the number of outcomes used. The continuous plaque score data available as mean and standard deviation (SD) for pre‐ and post‐brushing and incremental plaque scores were extracted as outcomes. Any data approximation by interpreting figures was avoided to ensure an accurate estimate. In the case of missing data or undetermined information, attempts were made to contact the first or corresponding author of the included publications for clarification or to retrieve additional data.

### 2.8 Data synthesis

In summary, a descriptive data presentation was used for all studies comparing the plaque score reduction from 1 min and 2 min of brushing duration. When feasible, a meta‐analysis was performed on the outcome plaque index scores. Based on methodological heterogeneity, it was decided a priori that MTB and PTB were to be evaluated separately as groups.

When the same plaque index scores were used, the difference of means (DiffM) was calculated. Otherwise, the standardized mean difference (SMD) was determined. If there were four or more comparisons to be analysed, the ‘random effects’ model was chosen to calculate the weighted average of the treatment effects across the studies. If there were fewer than four studies, the common effects model was used.^30^ The confidence intervals between the 1‐ and 2‐min brushing durations were calculated using (Review Manager (RevMan), version 5.4; for Windows, Copenhagen, Denmark: The Nordic Cochrane Centre, The Cochrane Collaboration, 2020).

### 2.9 Interpretation

A *p*‐value smaller than or equal to 0.05 (≤0.05) was considered statistically significant. Confidence intervals with little overlap were considered a strong indication of statistical heterogeneity. When studies were considered similar enough to allow for a comparison, their heterogeneity was statistically examined. The heterogeneity in the meta‐analysis was tested with the chi‐square test and the *I* ^2^ statistic.^31^, ^32^, ^33^ A chi‐square test resulting in a *p*‐value of <0.1 was considered an indication of significant statistical heterogeneity. As an approximate guide for assessing the degree of inconsistency across studies, an *I* ^2^ result of 0%–40% was interpreted as potentially unimportant, 30%–60% as representing moderate heterogeneity, 50%–90% as representing substantial heterogeneity and 75%–100% as representing considerable heterogeneity.^34^ The continuous measurement effect size SMD is known as Cohen's *d*. The magnitude of the effect was interpreted as small, 0.2; medium, 0.5 and large, 0.8.^35^

### 2.10 Publication bias

Funnel plots per performed meta‐analysis outcome were used as a formal detection of publication bias and were performed by a visual inspection of the plot for assessment, with a minimum of 10 comparisons.^36^ The asymmetry in the inverted funnel was suggestive of publication bias.^28^, ^37^

### 2.11 Grading the body of evidence

The Grading of Recommendations Assessment, Development and Evaluation (GRADE) was used to rank the evidence and certainty.^38^ The quality of the evidence and the strength and direction^39^ of the recommendations were graded for risk of bias, consistency of results, directness of evidence, precision and publication bias and the magnitude of the effect.

## 3 RESULTS

### 3.1 Search and selection results

**FIGURE 1:**
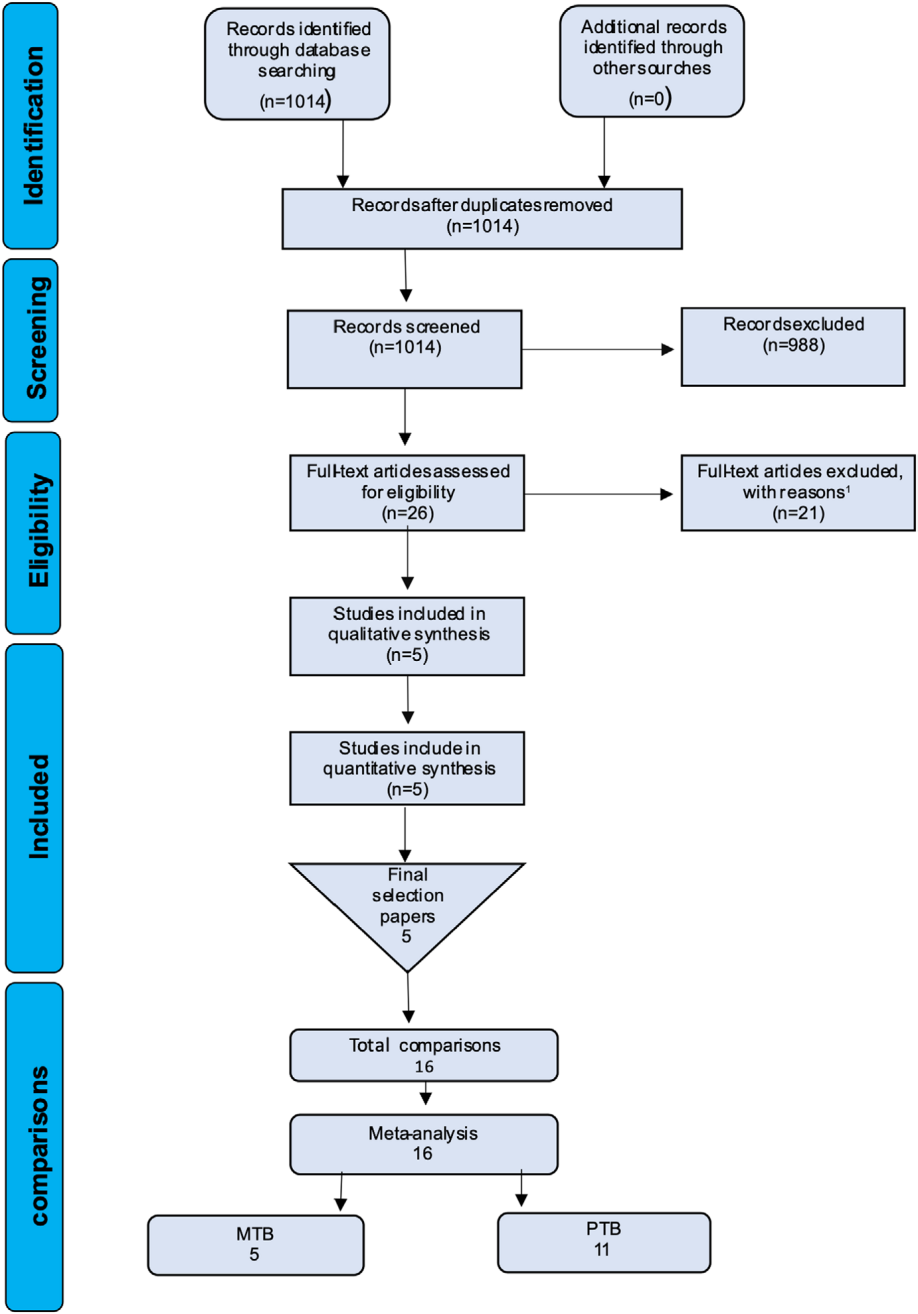
Flow chart of the search and selection process.

A total of 1014 unique papers were identified using the previously mentioned search strategy. Based on a screening of the titles and abstracts, 26 papers were selected, and their full texts were read. The Appendix S2 shows an overview of the rejected studies together with reasons for their rejection. Finally, five full‐text papers were included in the present systematic review.^13^, ^15^, ^40^, ^41^, ^42^ Multiple studies provided more than one comparison due to the use of several toothbrushes,^15^, ^42^ several plaque indices,^42^ brushing with or without dentifrice^13^ and different brushing forces.^41^ A manual search of the reference lists of these selected papers provided no additional suitable publications. Consequently, out of the five full‐text papers, 16 eligible comparisons were found and included in this systematic review (Figure 1). In total, 5 comparisons were used for the MTB, and 11 comparisons were used for the PTB.

### 3.2 Study characteristics and heterogeneity assessment

Information regarding the study design outline and characteristics is shown in detail in Table 2. All studies were RCT/CCTs with a parallel or a cross‐over design using different brands of MTBs and PTBs. Not all studies used a dentifrice or a familiarization period. Moreover, the instructions and brushing facilities differed among the studies (see Table 2 for further information). One study^41^ was originally designed to evaluate the effects of different brushing forces ranging from 75 to 300 g, and one study^13^ was originally designed to evaluate the effects of brushing with and without dentifrice.

**TABLE 2:** Characteristics of the included studies evaluating the comparison 1 and 2 min toothbrushing. An overview of the studies processed for data extraction.

Author year	Participants age	Toothbrush	Instruction	Familiarisation period	Conclusion of the original authors
Study design	Gender	Brand/type		Dentifrice (DF)	
	Country	MTB/PTB			
Gallagher et al. (2009) RCT Cross over	47 (46) ♀: 37 ♂: 10 Mean age: ? Age range: 18–63 USA	MTB Aquafresh Flex/ flat trim	Six times a single exercise No instruction prior Brushing under supervision	No 2 comparisons: 1 comparison DF:+ 1.5 g Aquafresh advanced 1 comparison DF: no	These results reinforce the view that oral health professionals, while coaching their patients in brushing technique should recommend brushing for at least 2 min
McCracken et al. (2003) RCT Cross over WoP: 3 days	12 (12) ♀:? ♂:? Mean age: ? Age range: 18–30 England	PTB Philips Jordan	4 consecutive days over four consecutive weeks. Brushing force (75, 150, 225, 300 g) was randomised for each week and the order of the brushing times, over the week, was randomised for each subject	Training and practice prior to trial. Home use Sensiflex to practice DF: ?	Brushing force and brushing time both significantly affect the level of plaque removed by a PTB. The effect was not uniform over the combinations of forces and times investigated
		PTB Sensiflex 2000			
Renton‐Harper et al. (2001) RCT ?? Cross over WoP: 2.5 days	16 ♀: 10 ♂: 6 Mean age: ? Age range 23–41 England	MTB OralB 35	Participants received 1 week before the study a PTB, no instructions were given	7 days habituation period PTB DF:Colgate Regular	Greatest proportion of plaque removal in the first 30 s and then incrementally less at subsequent brushing times
		PTB OralB D5			
		PTB OralB D9			
George et al. (2016) CCT Cross over WoP: ?	40 ♀: 30 ♂: 10 Mean age: ? Age range 18–65 India	MTB Colgate	No instruction prior to trial, brushing time was divided evenly between four quadrants.	No DF:Colgate Total	The results of the study clearly shows that brushing time in an important determinant in plaque removal
Van der Weijden et al. (1996) RCT Cross over WoP: ?	54 (49) ♀:? ♂:? Mean age: ? Age range: ? The Netherlands	PTB Braun Plak Remover (D7)	Three‐way cross‐over. The equivalent of 1 and 2 min in each quadrant Participants were guided through brushing exercises	Familiarisation for 2 weeks DF: Zendium at home. Standard toothpaste in brushing trial	A high level of efficacy is reached on the vestibular and lingual surfaces with the increase in brushing time
		PTB modified Plak Control (D9)			
		PTB Sonicare			

Abbreviations: CCT, controlled clinical trials; MTB, manual toothbrush; PTB, powered toothbrush; RCT, randomied controlled trials; WoP, Wash out Period: ?, unknown

### 3.3 Risk of bias assessment

The estimated potential risk of bias in the included studies was assessed (see Appendix S3 for further information). All the included studies were RCTs or CCTs with a randomly generated sequence, but none reported allocation concealment. None had selectively reported or presented incomplete outcome data. Only one study showed another form of potential bias, as authors were employed by the industry,^13^ and one study was unclear regarding the blinding of the outcome assessment.^40^ The estimated risk of bias for these two studies was moderate.^13^, ^40^ The other three studies indicated a low risk of bias.^15^, ^41^, ^42^

### 3.4 Study outcome results

#### 3.4.1 Data extraction

Appendix S4 shows the individual results from the data extraction of the plaque index scores from the selected publications.

#### 3.4.2 Descriptive analysis

Table 3 presents the descriptive qualitative analysis. There were 16 comparisons of 1‐ and 2‐min brushing durations. Data were presented for either an MTB (5x) or a PTB (11x). Of these comparisons, 60% with an MTBs and 91% of those with a PTB showed a statistically significant difference in favour of 2 min.

**TABLE 3:** A descriptive summary of statistical significance levels of the difference in 1 min compared to 2 min of brushing time for MTB and PTB.

a. MTB					
Author/year	Plaque index	Intervention		Significant between groups	Control
Renton‐Harper et al. (2001)	Q&H	1 min		+ ◊	2 min
	AS&M	1 min		+ ◊	2 min
George et al. (2016)	Q&H	1 min		+	2 min
Gallagher et al. (2009)	Q&H	DF +	1 min	0 ◊	2 min
		DF −	1 min	0 ◊	2 min
Overall: 5 comparisons, 3× −, 2× 0 (60% of the comparisons show a statistical significant difference in favour of 2 min)					

b. PTB					
Author/year	Plaque index	Brand	Intervention	Significant between groups	Control
Van der Weijden et al. (1996)	S&L	D7	1 min	+ ◊	2 mins
		D9	1 min	+ ◊	2 mins
		Sonicare	1 min	0 ◊	2 mins
Renton‐Harper et al. (2001)	AS&M	D5	1 min	+ ◊	2 mins
		D9	1 min	+ ◊	2 mins
Renton‐Harper et al. (2001)	TQ&H	D5	1 min	+ ◊	2 mins
		D9	1 min	+ ◊	2 mins
McCracken et al. (2003)	Q&H	75 g	1 min	+	2 mins
		150 g	1 min	+	2 mins
		335 g	1 min	+	2 mins
		75 g	1 min	+	2 mins
Overall: 11 comparisons, 10× −, 1× 0 (90.9% of the comparisons show a statistical significant difference in favour of 2 min)					

*Note*: DF+: with dentifrice; DF−: without dentifrice; AS&M: Addy et al. (1983)^24^ modification of Shaw&Murray (1977)^25^ (Yeung et al., 1983)^43^ (Smith et al., 2004)^44^; Q&H/TQ&H: Turesky (1970)^20^ modification of Quigley&Hein (1962)^19^ (Turesky, 1970)^20^; S&L: Silness et al. (1964) (Silness & Löe, 1964);^23^ ◊ : calculated by authors; − : intervention (1 min) is more effective than control (2 mins); + : control (2 mins) is more effective than intervention (1 min); 0: no significant difference between intervention and control.

#### 3.4.3 Meta‐analysis

Table 4 summarises the outcome details of the MA on the plaque index score data. The meta‐analyses were performed using a random effect model and presented as SMD due to the variety of plaque index scores used in the included papers. For detailed forest plots of the MA, see Appendix S5. Data are presented for the MTB groups (Figure 5A,B in Appendix S5) and PTB groups (Figure 5C,D Appendix S5). Appendix S5E,F shows the interpretation of the effect sizes. It was only possible to perform a meta‐analysis of the baseline and end data. It was impossible to analyse incremental differences among plaque scores, as the SDs were unavailable, even after approaching the original authors with a request to provide these.

**TABLE 4:** Meta‐analysis. (A) Summary of the meta‐analysis of the SMD in MTB For the primary parameters of interest. Presented for the baseline and the end scores using a random effect model. (B) Summary of the meta‐analysis of the SMD in PTB for the primary parameters of interest. Presented for the baseline and the end scores using a random effect model.

Measurement moment	Analysis	# Included studies	Model	SMD	Test overall		Test for heterogeneity		For details see the appendix
					95% CI	*p*‐Value	*I* ^2^ Value (%)	*p*‐Value	
Baseline	Participants brushing	# 5	Random	−0.03	(−0.30; 0.25)	0.84	0%	1.00	S5.B1
End	Participants brushing	# 5	Random	0.69	(0.06; 1.33)	**0.03**	76%	**0.002**	S5.B2

Measurement moment	Analysis	No. of Included studies	Model	SMD	Test overall		Test for heterogeneity		For details, see the Appendix
					95% CI	*p*‐Value	*I* ^2^ value (%)	*p*‐Value	
Baseline	Participants brushing	# 11	Random	−0.04	(−0.22; 0.15)	0.71	0%	1.00	S5.C1
End	Participants brushing	# 11	Random	0.47	(0.28; 0.66)	**<0.00001**	0%	0.96	S5.C2

*Note*: *p*‐Values are presented in bold if *p* ≤ 0.05. For interpretation colours of the SMD and *I* ^2^, see Appendix S5G–H.

Abbreviations: MTB, manual toothbrush; NA, not applicable; SMD, standardised mean difference.

There was no difference among the toothbrushes or subgroups for the baseline plaque scores. A heterogeneity of 0% was considered not important. The end‐point data of the MTB included 5 comparisons between 1 and 2 min of brushing duration with an overall standard mean difference (SMD) in favour of the 2 min of brushing duration of 0.69 [95%CI:0.06, 1.33], (*p* = 0.03). This effect can be interpreted as a medium effect,^35^ and the heterogeneity of 76% was estimated as substantial.^34^ The end scores for the PTB showed a significant effect in favour of 2 min of brushing, with an SMD of 0.47 [95% CI: 0.28, 0.66], (*p* < 0.00001), which also can be interpreted as a medium effect and the heterogeneity of 0%, which was considered unimportant.

Appendix S6 (Figure 6A,B) shows the funnel plots of the base and end scores for PTB. No publication bias seemed present. For MTB there were not enough studies to conduct funnel plots.

### 3.5 Evidence profile

Table 5 shows a summary of the various factors used to rate and assess the certainty of the quality of evidence and the strength of recommendations according to GRADE^38^, ^45^ The magnitude of the effect of brushing for 1 min versus 2 min on plaque score levels was medium. The strength of the evidence for this finding was estimated to be moderate. Consequently, 2 min of brushing can be recommended over 1 min of brushing with moderate certainty.

**TABLE 5:** Summary of the findings on the body of the estimated evidence profile and appraisal of certainty and the strength of the recommendation regarding 1 min compared to 2 min of brushing time on plaque removal.

Determinants of the quality	Overall
Study design	RCT
# Studies (Table 2, Figure 1)	5
# Comparisons (Figure 1)	16
# Meta‐analysis (Appendix S5)	16
Risk of bias (Appendix S3)	Low to moderate
Consistency (Appendix S5)	Rather consistent
Directness	Generalizable
Precision (Appendix S5)	Rather precise
Reporting bias (Appendix S6)	Possible
The magnitude of the effect^35^	Medium
Strength of the recommendation based on the quality and body of evidence	Moderate
**Overall recommendation**	**There is moderate certainty in support of the recommendation to brush for 2 min over 1 min of brushing for a medium more effective plaque removal**

Abbreviation: RCT, randomised controlled trial.

## 4 DISCUSSION

### 4.1 Summary of findings

This review provides an overview of 5 studies with 16 comparisons evaluating the effect of 1 and 2 min of brushing on plaque score reduction. Data from 328 participants published in the period from 1993 to 2016 showed limited methodological and clinical heterogeneity. It allowed for a descriptive qualitative and quantitative meta‐analysis with potentially substantial statistical heterogeneity. These analyses indicate, with moderate certainty, a modest difference in plaque scores resulting from brushing for 2 min instead of 1 min. Consequently, favouring the recommendation of 2 min over 1 min of toothbrushing.

### 4.2 Heterogeneity

The inclusion criteria for the present SR were strict enough to ensure limited methodological and clinical heterogeneity. Specifically selecting only clinical trials among adults using a single brushing exercise design. This selection also supports the finding that the statistical heterogeneity of the baseline scores was *I* ^2^ = 0% (see Table 4 and Appendix S5), which is potentially unimportant. Furthermore, limited heterogeneity can be the result of randomisation and selecting by comparable, balanced population of groups at baseline. A meta‐analysis of the end scores showed an *I* ^2^ of 0% for studies with a PTB and 76% for those with an MTB, which is estimated as having none‐to‐substantial statistical heterogeneity. It also supports the ‘a priori’ decision to consider MTB and PTB studies as separate study groups based on methodological and clinical heterogeneity.

### 4.3 Intervention and control

According to the *Cochrane Handbook*, a random effects meta‐analysis allows for study outcomes that vary from normal distribution among studies.^46^ The present SR used a random model based on that of the Cochrane Oral Group.^30^ Although the SR and the subsequent MA were based on 16 comparisons, these originated from five studies with minor methodological and clinical differences, thus explaining the limited statistical heterogeneity. A large data set is necessary for a thorough investigation of heterogeneity. From an epidemiological perspective, the 328 participants included in the present SR can be regarded as a limited number.^47^ Therefore, the effect sizes and accompanying confidence intervals should be interpreted with caution.

The present study compared the effect of 1 min and 2 min of brushing on plaque score reduction. These brushing durations were chosen because one represents the standard professional advice of 2 min of brushing, and the other intervention represents the more common practically performed 1‐min brushing duration by patients.^11^ Both brushing durations are effective for plaque removal, but the extent to which they are effective was questioned. For the present study, it could be argued that 2 min of brushing is the professional standard and should thus serve as the intervention, with the commonly used 1 min of brushing as the control. In dentistry, the consideration of choosing a control as intervention or vice versa is often the case, for instance, when comparing an MTB and a PTB, which are both effective oral hygiene devices.^9^, ^10^ For practical reasons in the SRs, the MTB is considered the control and the PTB the intervention as a MTB is seen as the basic procedure of toothbrushing.^9^, ^48^ However, it is generally accepted that the PTB is more effective than the MTB^49^, ^50^, ^51^, ^52^, ^53^ which also could imply that a PTB serves as active control and the MTB as intervention. This could also be the case when comparing the effect of toothpaste. Using toothpaste is commonly practised, in the SR evaluating the additional effect of toothpaste, the use of no toothpaste serves as the control and regular fluoride toothpaste is considered the intervention.^8^ Consequently, in some cases, the intervention and the control in PICO format are interchangeable. When searching for and selecting papers with specific intervention and control, there is a risk of not including potentially relevant papers that present the necessary comparison.

### 4.4 Single brushing and blinding

A single brushing exercise can be seen as a short‐term study on the efficacy of plaque removal and is considered proof of principle. To avoid influences such as nutrition, lifestyle, habits compliance, Hawthorne and novelty effects, the single‐use design can be used under ideal circumstances when only plaque removal outcome is of interest.^54^, ^55^ The perception of duration of brushing is complex; for instance, people believe that they brush for between 1 and 2 min, but the actual observed brushing duration is under 1 min.^11^, ^56^ Awareness of the intervention regimen may impact the performance and, consequently, the outcome measurement. This awareness may introduce a risk of overestimating the effect of the longer brushing duration. Therefore, long‐term evaluations of 1‐min to 2‐min comparisons are also of interest based on other influences.

It was an ‘a priori’ decision not to include ‘blinding the participants’ in the overall estimation of the risk of bias as brushing duration of 1 min or 2 min is easily discernible. The impossibility of blinding often occurs in oral health–related SRs. For instance, it is impossible to blind study participants to mechanical plaque control interventions. Participants use oral care products, such as toothbrushes and interdental cleaning devices, daily and know their specific features.^29^, ^57^

### 4.5 The perspective

Although brushing is a daily habit for most of the world's inhabitants, the effect of all factors contributing to its efficacy are still unknown.^58^ For instance, the amount of dental plaque accumulation necessary to induce gingival inflammation varies widely. In the case of severe periodontal disease, it is recommended not to exceed 10%–20% of dental plaque to avoid further progression.^3^ For most people, brushing is a difficult task. Thus, individually coaching people in using a toothbrush appropriately is important.^50^, ^51^, ^52^, ^53^ In addition, to be interested in learning the most effective brushing technique,^59^ people need help with motivation and compliance.^60^ The progress of plaque removal is not linear. It forms a smooth curve dependent on increasing brushing duration.^13^, ^42^ Therefore, the effectiveness of plaque removal after the first minute of brushing is greater than after the second minute. SRs on single brushing exercises support the finding that the time spent on brushing is important to the cumulative effect of plaque removal.^9^, ^10^ Although the effect size in the plaque score reduction of 2 min of brushing compared to that of 1 min of brushing is estimated to be medium, it is in support of the ‘professional standard’ of advice to brush for at least 2 min.^12^, ^61^ However, it remains uncertain what the oral health risk is when 1 min of brushing is practised in the long term. Adjusting the advice to 1 min of brushing may activate an ethical discussion because duration perception could result in even less brushing duration. It would be of interest to determine the effect of brushing for more than 2 min, for instance, by prolonging brushing duration by 1 min resulting in 3 min in total, compared to the standard advice of 2 min of brushing.^62^ This is a direction for further research by the use of a systematic review.

## 5 CONCLUSION

With respect to plaque scores, based on single brushing exercises, there is moderate certainty for the recommendation to brush for 2 min over 1 min. The clinical relevance of this difference can be estimated as medium.

## 6 CLINICAL RELEVANCE

### 6.1 Scientific rationale for the study

A systematic synthesis of the available data directly comparing 1 min of brushing duration with 2 min of brushing duration is currently lacking, although 2 min is accepted as the standard for toothbrushing duration.

### 6.2 Principal findings

After 2 min of brushing, the level of plaque scores is lower than after 1 min of brushing.

### 6.3 Practical implications

Both brushing durations appear effective, achieving a plaque score reduction of at least 13.4%. With moderate certainty 2 min of brushing duration can be recommended over 1 min of brushing duration. However, given the small difference, the clinical relevance can be estimated as medium.

## AUTHOR CONTRIBUTIONS

All authors gave their final approval and agreed to be held accountable for all aspects of the work, ensuring integrity and accuracy. **Marion T. Seuntjens**: contributed to conception and design, analysis, interpretation and drafted the manuscript. **Tim M. J. A. Thomassen**: contributed to analysis, interpretation and critically revised the manuscript. **Fridus (G.A.) Van der Weijden**: contributed to analysis, interpretation and critically revised the manuscript. **Dagmar Else Slot**: contributed to conception and design, analysis and interpretation and critically revised the manuscript.

## FUNDING INFORMATION

This research received no specific grant from any funding agency in the public, commercial or not‐for‐profit sectors. The work for this paper was funded by the regular academic appointments of Van der Weijden, Thomassen and Slot at the Academic Centre for Dentistry Amsterdam (ACTA). This paper was prepared as a part of the obligation of the first author Seuntjens to fulfil the requirements of the University of Amsterdam master's program in Evidence‐Based Practice in Health Care. Slot and Van der Weijden have previously received either external advisor fees, lecturer fees or research grants from toothbrush manufacturers. Those manufacturers included Colgate, Dentaid, GABA, Lactona, Oral‐B, Procter & Gamble, Philips, Sara Lee, Sunstar, Waterpik and Unilever. Van der Weijden has formerly received two unrestricted educational grants from Procter & Gamble Worldwide Clinical Investigations—Oral Care.

## CONFLICT OF INTEREST STATEMENT

The authors declare that they have no conflicts of interest.

## Supporting information


Appendix S1.

## Data Availability

The data that support the findings of this study are available in the supplementary material of this article.
