# Supplementary material for: Plaque scores after 1 or 2 minutes of toothbrushing A systematic review and meta‐analysis
Source: Int J Dent Hyg. 2025 Apr 8;23(3):614–24. doi: 10.1111/idh.12840 (PMC12371311; doi:10.1111/idh.12840)

**What is the effect of 2 compared to 1 minute brushing time on plaque removal?**

**=a systematic review and meta-analysis=**

**Marion T. Seuntjens (**https://orcid.org/0000-0003-1718-7695)

**Tim M. J. A. Thomassen** (https://orcid.org/0000-0003-2633-3236)

**Fridus (G.A.) Van der Weijden** (https://orcid.org/0000-0002-5075-8384)

**Dagmar Else Slot** (https://orcid.org/0000-0001-7234-0037)

**Online appendices**

**Supporting information**

Additional Supporting information may be found in the online version of this article.

**Appendix 1.**Ethical approval of the ACTA institute.

**Appendix 2.**Papers excluded after full text reading with details for rejection.

**Appendix 3**Methodological quality and potential risk of bias scores of the individual studies that were included for this review

**Appendix 4.**Data extraction of the included studies
4.A**.** Data table with mean (SD) scores for MTB base and end scores of the studies that were included ordered by index used and who brushes.
4.B Data table with mean (SD) scores for PTB base and end scores of the studies that were included ordered by index used and who brushes.

**Appendix 5.**Quantitative analysis of the meta-analysis for the primary parameters of interest. Presented for the baseline, and end scores. Presented for the use of a fixed model and random when appropriate.
5.A: MTB base scores
5.B. MTB end scores
5.C: PTB base scores
5.D. PTB end scores
5 E. SMD effect size interpretation
5 F. I^2^ value interpretation

**Appendix 6.**Funnel Plots of the meta-analysis for the primary parameters of interest on base and end scores.

**Appendix 1**

**Ethical approval of the ACTA institute.**


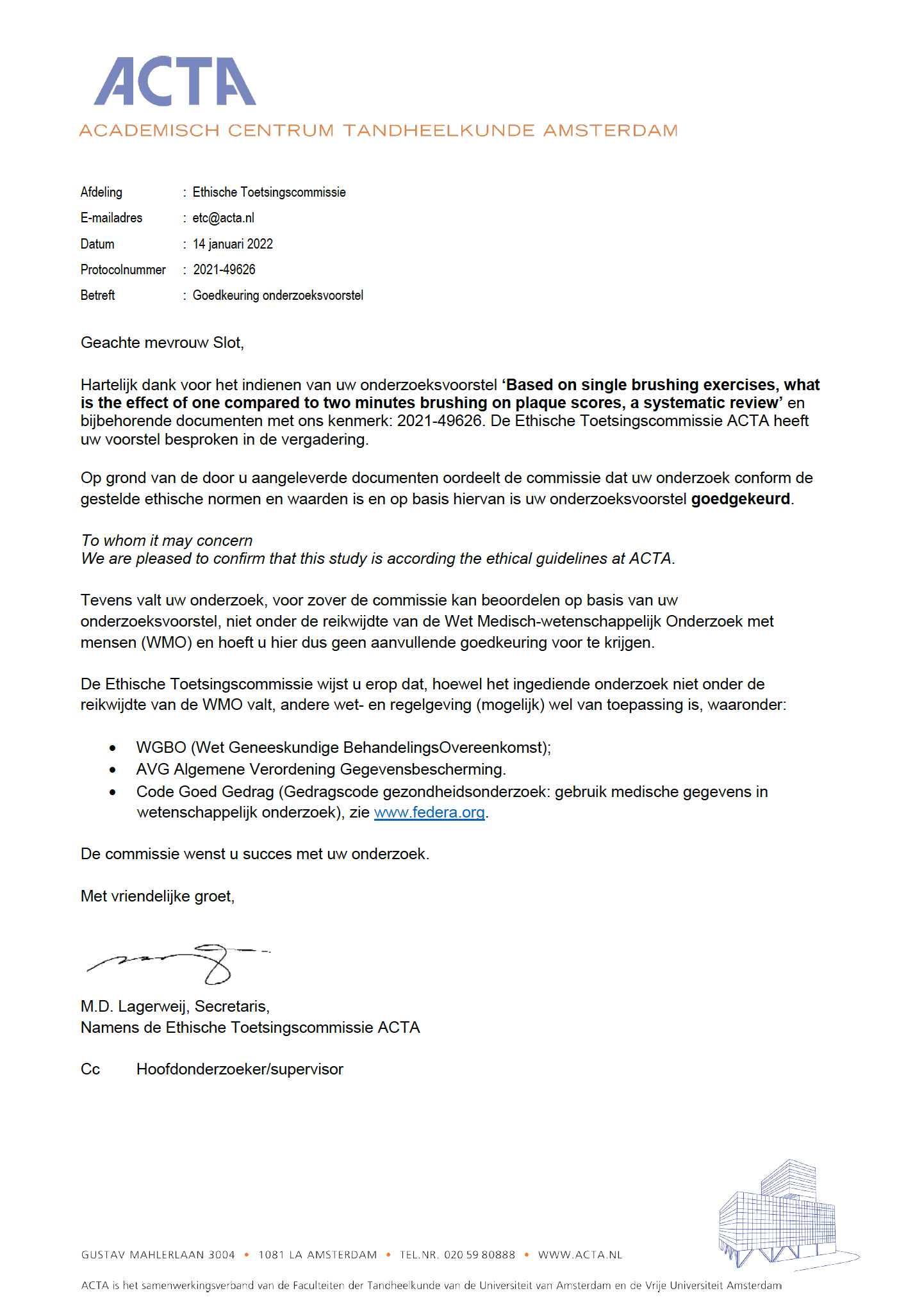


**Appendix 2**

**Papers excluded after full text reading with details for rejection.**

| **Author, Year** | **Reason for rejection** |
| --- | --- |
| Frandsen, 1972  Lang, 1973  Hawkins 1986  Dentino A. 2002  Davies et al. 2003  Muller-Bolla et al. 2012  Harnacke et al. 2015  Ganss et al. 2020 | No brushing time comparison |
| Preber et al. 1991  Saxer et al. 1998  Van der Weijden et al. 2004  Williams et al. 2004  McCracken et al. 2005  Van der Weijden et al. 2005  Muller-Bolla et al. 2007  Pelka et al. 2011  McKenzie et al. 2018 | Not the correct time comparison |
| Bergenholtz et al. 1969  Hansen, Gjermo, 1971 | No comparison of plaque levels and brushing time. |
| Terèzhalmy et al., 2008 | Compared flat trim to cross angled design bristle tuft configuration |
| Van der Weijden et al. 1993 | Professional brushing |

**Appendix 3
Methodological quality and potential risk of bias scores of the individual studies that were included for this review**

|  | | Random sequence generation (selection bias) | Allocation concealment  (selection bias) | Blinding of participants or personnel (performance bias)al | Blinding of outcome assessment (detection bias) | Incomplete outcome data  (attrition bias) | Selective reporting  (reporting bias) | Others | Overall Risk of Bias |
| --- | --- | --- | --- | --- | --- | --- | --- | --- | --- |
|  | Van der Weijden et al. 1996 | Y | N | NA | Y | Y | Y | Y | low |
|  | Renton-Harper et al. 2001 | Y | N | NA | Y | Y | Y | Y | low |
|  | McCracken et al. 2003 | Y | N | NA | Y | Y | Y | Y | low |
|  | Creeth et al. 2009 | Y | N | NA | Y | Y | Y | U | moderate |
|  | George et al., 2016 | Y | N | NA | U | Y | Y | Y | moderate |

NA: not applicable.

Y:Yes

N:No

U: Unclear

**Appendix 4**

**Data extraction of the included studies**

4.A. Data table with mean (SD) scores for MTB base and end scores of the studies that were included ordered by index used.

| **Author (year)** | **Index** | **Brand** | **brushing duration** | **Baseline** Mean (SD) | **End**  Mean (SD) | **Difference** | |
| --- | --- | --- | --- | --- | --- | --- | --- |
|  |  |  |  |  |  | Mean | % |
| Renton-Harper et al. 2001 | AS&M^a^ | Oral-B 35 | 1 minute | 1.19 (0.19) | 0.57 (0.16) | 0.62 ◊ | 51.9 |
|  |  |  | 2 minutes | 1.186 (0.188) | 0.425 (0.126) | 0.76 ◊ | 64.2 |
|  | TQ&H^b^ | Oral-B 35 | 1 minute | 3.179 (0.208) | 2.020 (0.417) | -1.16 ◊ | 36.5 |
|  |  |  | 2 minutes | 3.18 (0.21) | 1.67 (0.36) | -1.51 ◊ | 47.5 |

| Gallagher et al. (2009) | TQ&H^b^ | Aquafresh Flex flat-trim soft toothbrush | | 1 minute | 2.95 (0.49) | 2.14 (0.57) | -0.81 ◊ | 27.5 ◊ |
| --- | --- | --- | --- | --- | --- | --- | --- | --- |
|  |  |  |  | 2 minutes | 2.98 (0.47) | 2.06(0.54) | -0.92 ◊ | 30.9 ◊ |
|  |  | Aquafresh Flex flat-trim soft toothbrush | DF- | 1 minute | 2.98 (0.53) | 2.13 ((0.60) | -0.85 ◊ | 28.5 ◊ |
|  |  |  | DF+ | 2 minutes | 2.98 (0.47) | 2.06(0.54) | -0.92 ◊ | 30.9 ◊ |
| George et al., 2016 | TQ&H | Colgate Total | | 1 minute | 3.06(0.06) | 2.37(0.07) | -0.69 | 22.6◊ |
|  |  |  |  | 2 minutes | 3.06(0.08 | 2.26(0.08) | -0.80 | 23.2◊ |

4.B. Data table with mean (SD) scores for PTB base and end scores of the studies that were included ordered by index used.

| **Author (year)** | **Index** | **Brand** | **Group (brushing duration)** | **Baseline**  Mean (SD) | **End**  Mean (SD) | **Difference** | |
| --- | --- | --- | --- | --- | --- | --- | --- |
|  |  |  |  |  |  | **Mean** | **%** |
| McCracken et al. (2003) | Q&H^e^ | Modified Sensiflex2000 | 1 minute (75g) | 3.89(0.33)⬥ | 3.37(0.44) ⬥ | -0.52 ◊ | 13.4 ◊ |
|  |  |  | 2 minutes (75g) | 3.90(0.30) ⬥ | 3.25(0.40) ⬥ | -0.65 ◊ | 16.6 ◊ |
|  |  |  | 1 minute (150g) | 4.00(0.26) ⬥ | 3.43(0.50) ⬥ | -0.57 ◊ | 14.3 ◊ |
|  |  |  | 2 minutes (150g) | 4.02(0.18) ⬥ | 3.02(0.41) ⬥ | -1.00 ◊ | 24.8 ◊ |
|  |  |  | 1 minute (225g) | 3.83(0.31 ⬥ | 3.05(0.50) ⬥ | -0.78 ◊ | 20.3 ◊ |
|  |  |  | 2 minutes (225g) | 3.88(0.23) ⬥ | 2.82(0.48) ⬥ | -1.06 ◊ | 27.3 ◊ |
|  |  |  | 1 minute (300g) | 3.89(0.27) ⬥ | 3.11(0.34) ⬥ | -0.78 ◊ | 20.0 ◊ |
|  |  |  | 2 minutes (300g) | 4.00(0.21) ⬥ | 2.90(0.50) ⬥ | -1.10 ◊ | 27.5 ◊ |
| Renton-Harper et al. 2001 | AS&M^a^ | Braun/Oral-B D5 | 1 minute | 1.18 (0.12) | 0.66 (0.21) | -0.52 ◊ | 44.1 |
|  |  |  | 2 minutes | 1.18 (0.12) | 0.49 (0.20) | -0.69 ◊ | 58.7 |
|  |  | Braun/Oral-B D9 | 1 minute | 1.19 (0.22) | 0.62 (0.17) | -0.57 ◊ | 48.1 |
|  |  |  | 2 minutes | 1.19 (0.22) | 0.45 (0.156) | -0.74 ◊ | 62.3 |
|  | TQ&H | Braun/Oral-B D5 | 1 minute | 3.14 (0.15) | 2.10 (0.43) | -1.04 ◊ | 33.2 |
|  |  |  | 2 minutes | 3.14 (0.15) | 1.74 (0.47) | -1.40 ◊ | 44.7 |
|  |  | Braun/Oral-B D9 | 1 minute | 3.21 (0.24) | 2.13 (0.47) | -1.07 ◊ | 33.5 |
|  |  |  | 2 minutes | 3.21 (0.24) | 1.78 (0.48) | -1.43 ◊ | 44.6 |
| Van der Weijden et al. (1996) | S&L^c^ | Braun Plaque remover | 1 minute | 1.90 (0.19) | 0.84 (0.45) | -1.06 ◊ | 56 |
|  |  |  | 2 minutes | 1.91 (0.15) | 0.59 (0.37) | -1.32 ◊ | 70 |
|  |  | modified Plak Control (D9) | 1 minute | 1.94 (0.12) | 0.80 (0.41) | -1.14 ◊ | 59 |
|  |  |  | 2 minutes | 1.94 (0.12) | 0.61 (0.34) | -1.33 ◊ | 69 |
|  |  | Sonicare | 1 minute | 1.94 (0.10) | 0.84 (0.47) | -1.10 ◊ | 57 |
|  |  |  | 2 minutes | 1.93 (0.17) | 0.75 (0.45) | -1.18 ◊ | 62 |

a:Addy et al. (1983) modification of Shaw&Murray (1977)
b: Turesky et al (1970) modification of Quigley&Hein (1962)
c: Silness et al (1964) (Silness&Loe Plaque Index.)
d: Rustogi et al (1992 )modification of the Navy Plaque Index
e. Quigley&Hein modification McCracken et al. 2002
(…g)= brushing force used in gram

◊ data calculated by the authors of this systematic review

⬥ data obtained from the original authors of the included paper

**Appendix 5.
Forrest Plots of the meta-analysis for the primary parameters of interest. Presented for the baseline, and end scores of MTB and PTB. Presented for the use of a fixed model and random when appropriate**

5.A: MTB base scores of difference in dental plaque levels before brushing 1 or 2 minutes

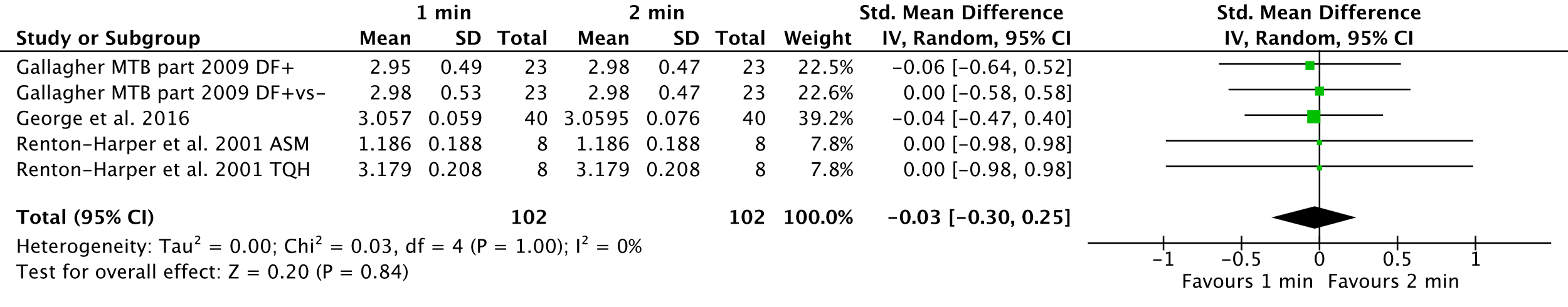


5.B: MTB end scores of difference in dental plaque levels after brushing 1 or 2 minutes

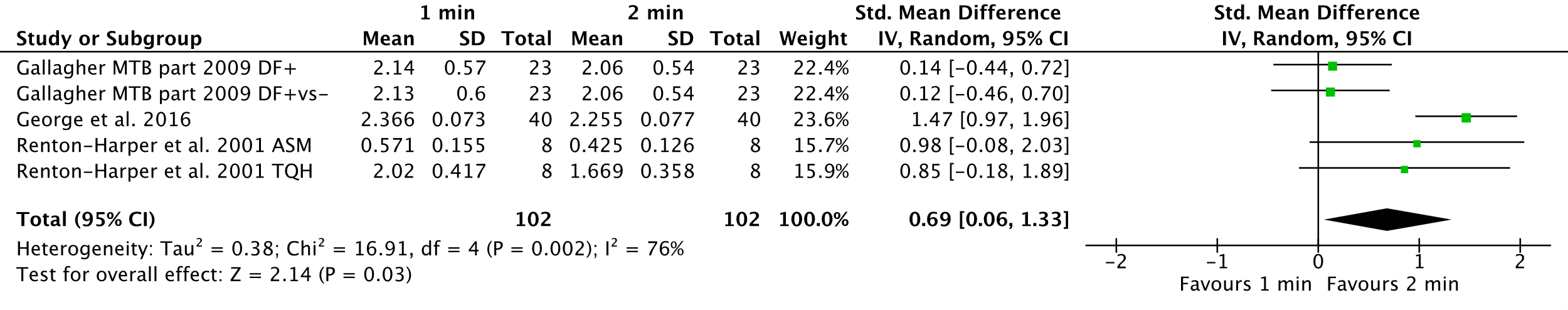


5.C. PTB base scores of difference in dental plaque levels before brushing 1 or 2 minutes

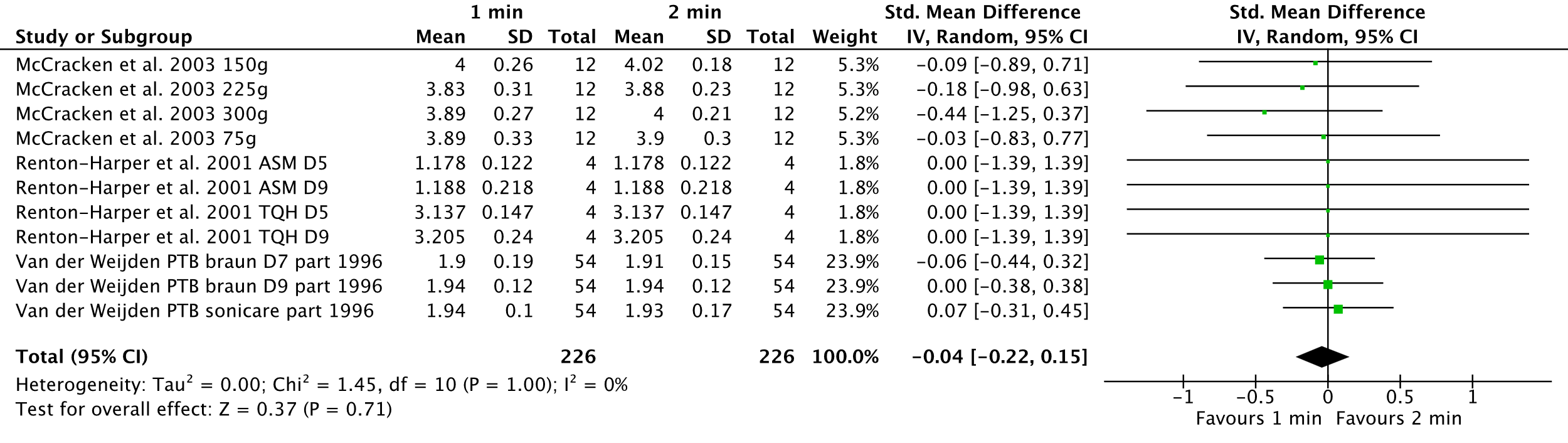


5.D. PTB end scores of difference in dental plaque levels after brushing 1 or 2 minutes

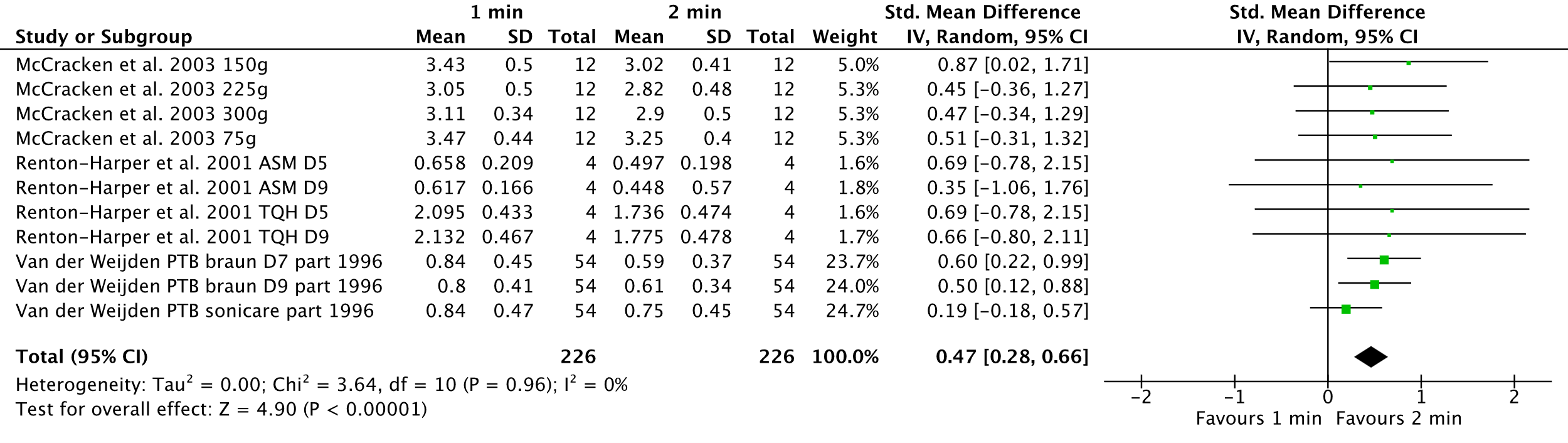


5.E. SMD effect size interpretation (Cohen, 1988)

| **None**  0-0.2 | **Small**  >0.2 - ≤0.5 | **Medium**  >0.5- ≤0.8 | **Large**  >0.8 |
| --- | --- | --- | --- |

5.F. I^2^ value interpretation(Higgins et al. 2022)

| **Potential not important**  0-40% | **Moderate**  30-60% | **Substantia**l  50-90% | **Considerable**  75-100% |
| --- | --- | --- | --- |

**Appendix 6.
Funnel Plots of the meta-analysis for the primary parameters of interest on base and end scores.**

6.A: PTB base scores


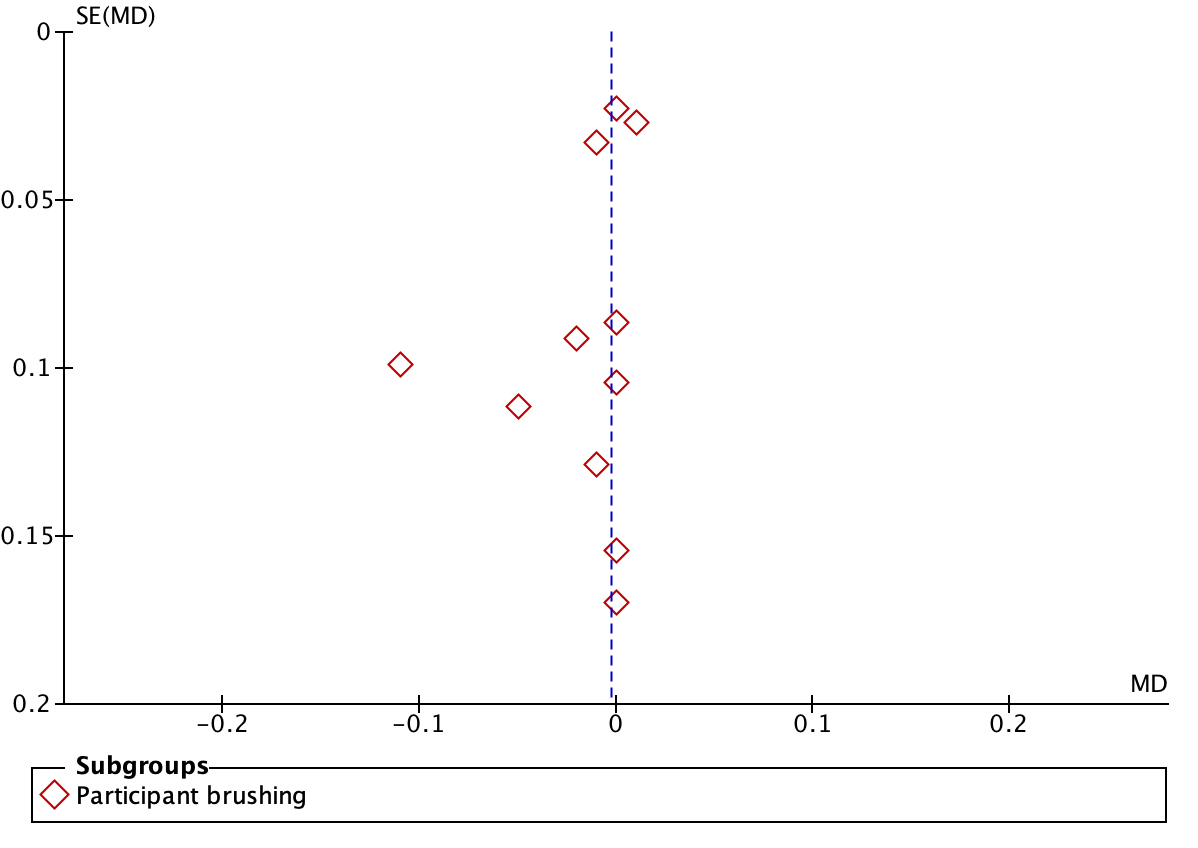


6.B: PTB end scores


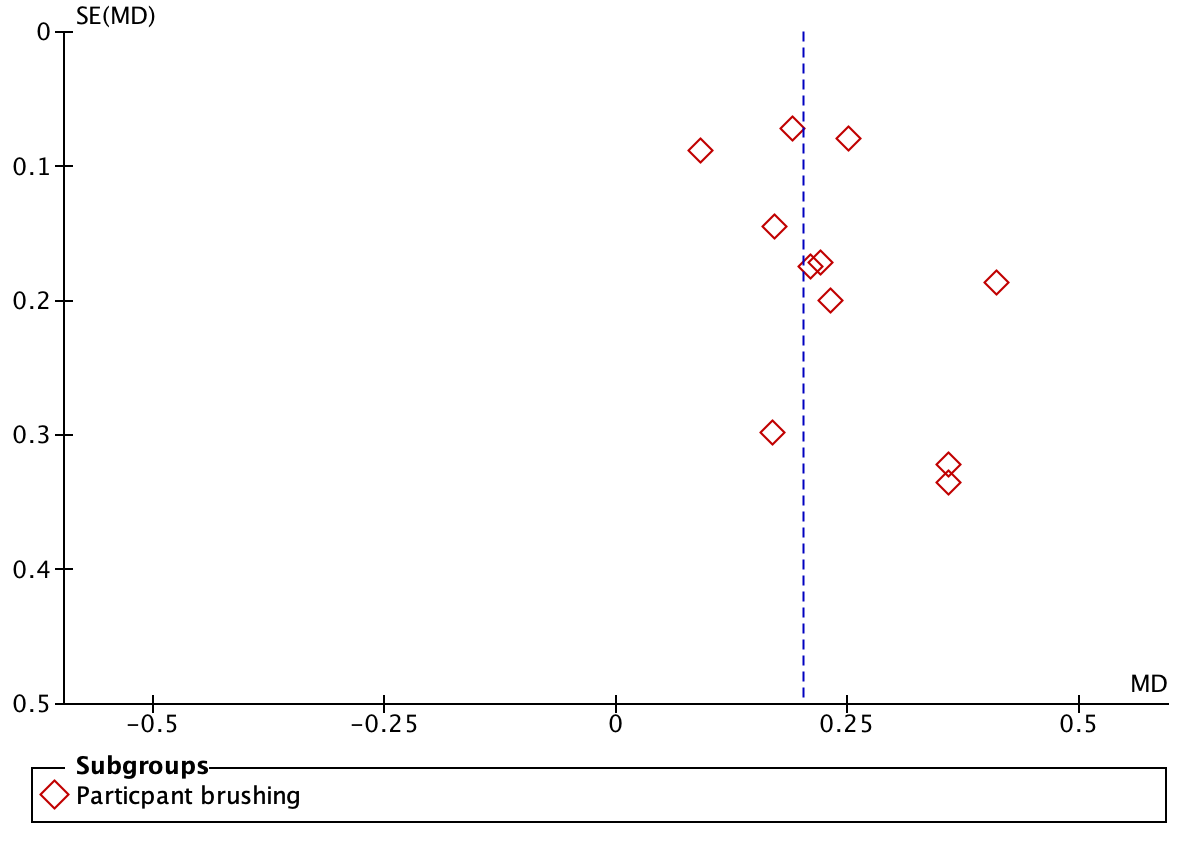

Supplement: Supplementary file 1 — Appendix S1. [file IDH-23-614-s001.docx]
